# Sustainable Development: Empowering Indigenous Peoples

**DOI:** 10.1289/ehp.113-a588

**Published:** 2005-09

**Authors:** Rebecca Kessler

Deforestation, erosion, and loss of biodiversity all directly affect Central American indigenous peoples’ sustenance, health, and way of life. The new Integrated Ecosystem Management in Indigenous Communities Regional Program (IEM) aims to alleviate these problems and the extreme poverty of many indigenous groups by helping communities manage their lands sustainably. The IEM will help communities establish and manage conservation areas and finance income-generating projects like sustainable tourism, sustainable forestry, and production of handicrafts, organic coffee and cocoa, and other traditional products. The program emphasizes traditional land management practices to combat declining bio-diversity, soil, and water quality.

“One objective is to strengthen local groups to prepare strategies to help with these problems,” says Alberto Chinchilla, regional facilitator for the Central America Indigenous and Peasant Coordination Association for Community Agroforestry. This group, along with the Central American Indigenous Council and Central American Commission for Environment and Development (CCAD), will implement the program.

The IEM will support small projects in some 550 communities in Belize, Costa Rica, El Salvador, Guatemala, Honduras, Nicaragua, and Panama, where nearly 7 million indigenous people account for about a quarter of the population. The Global Environment Facility granted the program $9 million through the Inter-American Development Bank (IDB) and the World Bank, cofinanced through other projects from both banks. Indigenous groups and CCAD will contribute another $2.5 million.

Indigenous groups’ environmental problems stem from their tenuous rights to the land they occupy, advocates say. Not all of the region’s nations enforce or even legally recognize indigenous land rights. Nor do governments or the World Bank require indigenous groups’ consent before approving projects that affect their lands or require their forced relocation, say advocates.

Poverty and encroachment onto their lands by ranchers, farmers and loggers wielding environmentally devastating practices has led many indigenous groups to forsake traditional land use practices for often unsustainable hunting, agriculture, and timber harvest methods. In agriculture, for instance, practices such as letting plots lay fallow for years at a time, intensive hand weeding, and cultivating a diversity of crops, often in the shade of fruit- or lumber-producing trees have in certain places given way to shorter crop rotation cycles, increased chemical use, and crop monocultures. The new practices may offer bountiful harvests in the short term, but they ultimately degrade the soil and are expensive to perpetuate. Struggling communities may also sell off timber or land for negligible sums to outsiders, who then clear the land for agricultural use, according to IEM documents.

Indigenous groups’ political influence is growing. But they continue to suffer from worse poverty, more disease, greater discrimination, and less education than other sectors of society, the World Bank concluded in its May 2005 report *Indigenous Peoples, Poverty and Human Development in Latin America: 1994–2004*.

Some indigenous activists question the IEM. “How will the [program] create sustainable development if the majority of the governments in the region don’t recognize the ability of indigenous communities to administer their lands, territories, and natural resources?” asks Hector Huertas, a lawyer from the Kuna tribe with the Centro de Asistencia Legal Popular, an indigenous advocacy group. He and others cast a wary eye on the World Bank and the IDB, whose projects, they say, typically leave a heavy environmental and cultural footprint. Many also believe the agencies charged with implementing the IEM may not truly represent indigenous peoples’ interests.

Some even argue that drawing indigenous communities into the cash economy through development projects threatens their autonomy. Rudolph C. Rÿser, chairman of the Center for World Indigenous Studies in Washington and a Cowlitz tribe member, says communities that provide for their own needs best exemplify sustainability. “People can say it’s unrealistic for indigenous communities to take care of themselves as autonomous economic units. They’d better realize it’s been going on for fourteen thousand years.”

## Figures and Tables

**Figure:**
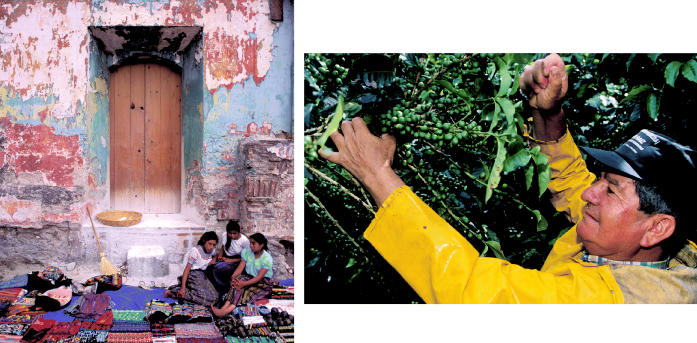
Sustaining themselves. Street vendors sell weavings in Guatemala (left), and a laborer picks coffee beans in Costa Rica (above). A new Central American project will finance such sustainable money-making activities to help indigenous groups prosper.

